# Dysfunctional eating behavior in fibromyalgia and its association with serum biomarkers of brain plasticity (BDNF and S100B): an exploratory study

**DOI:** 10.20945/2359-3997000000406

**Published:** 2021-09-29

**Authors:** Jéssica Lorenzzi Elkfury, Luciana C. Antunes, Letícia dal Moro Angoleri, Raquel Busanello Sipmann, Andressa de Souza, Iraci Lucena da Silva Torres, Wolnei Caumo

**Affiliations:** 1 Universidade Federal do Rio Grande do Sul Programa de Pós-graduação em Ciências Médicas Faculdade de Medicina Porto Alegre RS Brasil Programa de Pós-graduação em Ciências Médicas, Faculdade de Medicina, Universidade Federal do Rio Grande do Sul, Porto Alegre, RS, Brasil; 2 Universidade Federal do Rio Grande do Sul Faculdade de Medicina Porto Alegre RS Brasil Faculdade de Medicina, Universidade Federal do Rio Grande do Sul, Porto Alegre, RS, Brasil; 3 Universidade Federal de Santa Catarina Divisão de Nutrição Centro de Ciências da Saúde Florianópolis SC Brasil Centro de Ciências da Saúde, Divisão de Nutrição, Universidade Federal de Santa Catarina, Florianópolis, SC, Brasil; 4 Universidade Federal do Rio Grande do Sul Laboratório de Dor e Neuromodulação Porto Alegre RS Brasil Laboratório de Dor e Neuromodulação, Universidade Federal do Rio Grande do Sul, Porto Alegre, RS, Brasil; 5 Programa de Pós-graduação em Saúde e Desenvolvimento Humano Canoas RS Brasil Programa de Pós-graduação em Saúde e Desenvolvimento Humano, LaSalle, Canoas, RS, Brasil; 6 Universidade Federal do Rio Grande do Sul Programa de Pós-graduação em Ciências Biológicas: Farmacologia e Terapêutica Porto Alegre RS Brasil Programa de Pós-graduação em Ciências Biológicas: Farmacologia e Terapêutica, Universidade Federal do Rio Grande do Sul, Porto Alegre, RS, Brasil; 7 Universidade Federal do Rio Grande do Sul Instituto de Ciências Básicas da Saúde Departamento de Farmacologia Porto Alegre RS Brasil Departamento de Farmacologia, Instituto de Ciências Básicas da Saúde, Universidade Federal do Rio Grande do Sul, Porto Alegre, RS, Brasil; 8 Universidade Federal do Rio Grande do Sul Faculdade de Medicina Departamento de Departamento de Cirurgia Porto Alegre RS Brasil Departamento de Departamento de Cirurgia, Faculdade de Medicina, Universidade Federal do Rio Grande do Sul, Porto Alegre, RS, Brasil

**Keywords:** Fibromyalgia, eating behavior, eating disorder, BDNF, S100B

## Abstract

**Objectives:**

To assess disordered eating, hunger and satiety perceptions in women with fibromyalgia (FM) compared to healthy controls (HC) and their association with biomarkers of brain plasticity (brain-derived neurotrophic factor (BDNF) and S100 calcium-binding protein B (S100B)).

**Subjects and methods:**

Cross-sectional exploratory study. The sample included FM (n = 20) and HC (n = 19), matched to age and waist perimeter. Dysfunctional eating was assessed through the Three Factor Eating Questionnaire and Eating Disorders Examination with a questionnaire. Hunger and satiety levels were rated by a Numerical Scale. Serum leptin, S100B and BDNF were analyzed.

**Results:**

The MANCOVA analysis showed that the mean of Emotional Eating rates was 30.65% higher in FM compared to HC ( *p* = 0.015). Eating, shape and weight concerns were 77.77%, 57.14% and 52.22% higher in FM ( *p* = <0.001) compared to HC, respectively. Moreover, the FM group reported higher scores for feeling of hunger “[5.2 (±2.9) vs. 4.8 (±2.0); *p* = 0.042] and lower scores for satiety [7.0 (±1.7) *vs* . 8.3 (±1.0); *p* = 0.038]. In the FM group, serum BDNF was negatively associated with hunger (r = - 0.52; *p* = 0.02), while S100B was positively associated with hunger scores (r = 0.463; *p* = 0.004).

**Conclusion:**

The present findings support the hypothesis that the association between FM and obesity can be mediated by a hedonistic pathway. Further research is needed.

## INTRODUCTION

Fibromyalgia (FM) is a pain syndrome characterized by widespread chronic musculoskeletal pain with a dysfunction of the neuroimmune-endocrine systems ( 1 , 2 ). The clinical symptoms involve cognitive dysfunction, depressive symptoms, sleep disturbances, and pain catastrophizing behavior ( 1 , 2 ). The estimated prevalence range from 0.2% to 4.7% in the general population and it is about 2.0% in Brazil ( 3 ). Moreover, it has been demonstrated that the comorbidity between FM and overweight/obesity is estimated at 62% to 73%, which is higher than the percentage value found in the general population ( 4 ), which is about and 39% ( 5 ) and in Brazil 55,7% ( 6 ). Indeed, body weight excess was negatively associated with quality of life and positively related to pain ( 7 ), evidencing that the presence of obesity undermines the curse and prognosis of FM ( 8 ).

Several factors can be connected to the association of FM and obesity, such as side effects of pharmacological treatment, movement fear-avoidance (culminating in the reduction of locomotion activity) ( 9 ), frustration over functional limitation, biomechanical/structural changes, chronic stress (autonomic changes leading to disruption of the hypothalamic-pituitary-adrenal axis (HPA axis) and sleep-wake cycle disturbances ( 10 ), thyroid disease ( 11 ), inflammatory mediators ( 12 ), psychiatric and psychological comorbidities ( 13 ), and lifestyle issues related to diet ( 14 ) and sedentary lifestyle ( 15 ).

Furthermore, not only chronic pain but also obesity may be understood as a phenomenon of maladaptive neuroplasticity mediated by factors, such as brain-derived neurotrophic factor (BDNF) and S100 calcium-binding protein B (S100B) ( 16 ). Both of them have a relevant role in the pathophysiology of FM, in terms of the progression and maintenance of clinical features of this painful syndrome ( 1 , 16 ). Moreover, BDNF and S100B also play a role in obesity. In fact, it has been postulated that BDNF might perform functions related to satiety induction and increased energy expenditure in obesity ( 17 ), while S100B seems to be associated with inflammatory markers, and positively associated with body mass index (BMI), and with serum leptin levels ( 18 ). Since BDNF and S100B protein are involved in both FM and obesity, it is reasonable to hypothesize an interplay among these biomarkers as far as brain plasticity is concerned, in the modulation of eating behavior in FM. Nevertheless, although it is known that FM is related to disruption in the HPA axis, which can result in disruption in homeostatic eating, it is likely that different factors, such as alterations in neuroplasticity factors, higher levels of depressive symptoms and pain catastrophizing, might as well contribute to dysfunctional eating behaviors in FM ( 8 ).

Therefore, in this exploratory study, we tested the hypothesis that FM patients would present disrupted eating behavior compared to healthy participants matched to age and waist perimeter. Besides, we hypothesize that the dysfunction of eating behavior is related to the serum biomarkers of neuroplasticity (BDNF and S100B protein). Thus, this study was designed to achieve the following objectives: i) to compare the domains of eating behavior assessed by the Three Factor Eating Questionnaire (TFEQ-21) and eating disorder symptomatology through the Eating Disorder Examination Questionnaire (EDE-Q) in FM and healthy subjects; ii) to examine the relationship between the role of eating behavior with the serum BDNF and the serum S100B protein.

## MATERIALS AND METHODS

This exploratory study was conducted at *Hospital de Clínicas de Porto Alegre* (Rio Grande do Sul, Brazil) from 2016 to 2017. The protocol was reviewed and approved by the Ethics Committee Board of the *Hospital de Clínicas de Porto Alegre* (CAAE: 44071215.8.0000.5327). All subjects gave their written informed consent before participation. The hypotheses of this study were specified before the data were collected.

### Design overview, setting, and participants

The participants were screened at a public institution. For both groups the inclusion criteria were women aged between 18 and 65 years with BMI between 18.5-40 kg/m². The FM group was recruited from local community care units, an institutional chronic pain clinic, by referrals from other hospital units and by phone. FM was diagnosed according to the American College of Rheumatology criteria for fibromyalgia ( 19 ). A physician with more than 15 years of experience in a pain clinic, and highly skilled at diagnosing chronic pain conditions, re-examined patients and then re-confirmed their diagnosis. FM patients were included if they had experienced pain scored with a visual analog scale (VAS) ≥ 40 mm (i.e., moderate or severe pain). Additionally, the pain had to be associated with disability, as assessed by an affirmative answer to dichotomous questions (yes/no) of a structured questionnaire. The queries inquired if their pain had interfered with (I) work; (II) enjoyable activities; (III) responsibilities at home; (IV) relationships; (V) personal goals; and (VI) thinking clearly, problem solving, concentrating, or recall. Additional exclusion criteria were decompensated systemic diseases and thyroid diseases.

**Figure 1:**
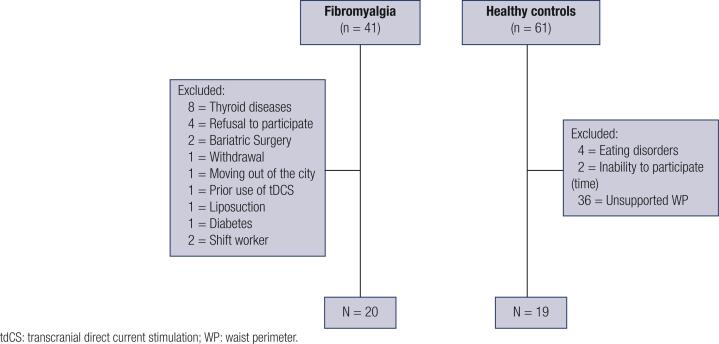
Flow diagram of study screening.

Healthy controls were recruited from the general population using public postings as well. A standard screening questionnaire was applied to assess inclusion criteria, which was set at age and waist perimeter, matched to the FM group. Eligible healthy control subjects had to be free of any acute or chronic pain, as well as any endocrinology, cardiovascular, rheumatologic, psychiatric, or neurological disorders, and without recent use of analgesics, corticosteroids or medications with known effects on the central nervous system as well as on metabolism. Exclusion criteria for both groups were pregnancy, shift work, people engaged in any kind of diet in the six months before the screening or people who had bariatric surgery or liposuction at any time in life. Furthermore, we have excluded patients who have been submitted to Transcranial Direct Current Stimulation (tDCS), a non-invasive neuromodulatory treatment, used to pain treatment in clinical research, since it could be a confounding variable due to its possible effect on the outcome variables ( Figure 1 ).

### Outcomes and measurements

The primary outcome was the domains of eating behavior, accessed by the Three Factor Eating Questionnaire (TFEQ-21) and the Eating Disorder Examination Questionnaire (EDE-Q). The TFEq-21 is a self-assessment scale that was designed to assess three cognitive and behavioral domains of eating: emotional eating, (EE) which is the susceptibility to eat in response to mood states, uncontrolled eating (UE), which is a tendency to lose control by overeating, even in the absence of physiological hunger, and cognitive restraint (CR), which is the limitation of food intake to control body weight ( 20 ). The EDE-Q is a self-report instrument for the assessment of disordered eating that can be a support for the diagnosis of eating disorders. The instrument generates a global score, which is the average of its four subscales scores, namely, restraint, eating concern, shape concern and weight concern ( 21 ).

The second outcome was serum biomarkers (BDNF and S100B). Leptin and estradiol were measured as confounders. Blood samples were collected in the morning (8-10 a.m.) after a 12-h fasting period. The blood samples were placed in plastic tubes and centrifuged for 10 minutes at 4,500 rpm at 4°C. Serum was stored at −80 °C for further assay. Serum mediator concentrations were determined using BDNF (Chemicon/Millipore, catalog no. CYT306, lower detection limit of the kit = 7.8 pg/mL), S100B (Millipore, Missouri, USA, catalog no. EZHS100B-33K, lower detection limit of the kit = 2.7 pg/mL), LEPTIN (Millipore, Missouri, USA, catalog no. EZHL-80SK, lower detection limit of the kit = 0.5 ng/mL) enzyme-linked immunosorbent assay (ELISA) kits, according to the manufacturers’ instructions. Estradiol was directly analyzed by the pathology service at HCPA.

### Other instruments and assessments

#### Sociodemographic questionnaire

The assessed variables were age, sex, work status, marital status and education.

#### Eating measures

We measured hunger, feeling of hunger, satiety and appetite to specific (sweet and savory) and unspecific foods on a 10-point numerical rating scale [from 0 (any desire or need to eat) to 10 (very prominent desire or need to eat)], according to a previous protocol ( 22 ). The participants were instructed to rate six questions about their level of: hunger, feeling of hunger, satiety, appetite for unspecific foods, appetite for sweet foods and appetite for savory foods. To check for a possible difference between feeding pattern and appetite pattern, we also asked subjects to report the time within a 24h period in which these measures were more and less manifested.

#### International Physical Activity Questionnaire-Long form (IPAQ-L)

The IPAQ-L asks details about the specific types of activities undertaken within each of the four domains (work, transport, domestic and garden, and leisure) ( 23 ). Total scores were calculated for walking, moderate-intensity activities and vigorous-intensity activities for an overall total.

#### Physiological measurements

*Brazilian Portuguese-Central Sensitization Inventory (BP-CSI):* applied to assess central sensitization related symptoms ( 24 ). Depressive symptomatology was assessed using the *Beck* *Depression Inventory II (BDI – II)* ( 25 ). A psychiatric evaluation was performed using the *Mini International Neuropsychiatric Interview – MINI 5.0* ( 26 ) to exclude psychiatric disorders in healthy participants. Sleep quality was measured using *the Pittsburgh Sleep Quality Index* ( 27 ).

#### Anthropometric measurements: body weight, height, BMI and waist perimeter

Height was measured in centimeters (cm) using a height scale (Sanny, 14024) with the subject standing barefoot and with a normal straight posture. Weight was measured in kilograms (kg) using a weight scale (Toledo, 2096 PP). BMI was calculated as the ratio of weight (kilogram) to the square of height (meters). Obesity and overweight were classified according to WHO criteria. A person was considered obese if their BMI value was ≥ 30 kg/m^2^, and overweight if BMI ≥ 25 kg/m^2^. Waist perimeter was measured in centimeters at the midpoint between the lower rib and the iliac crest using a non-stretchable measuring tape and were performed 3 times to a precision of 0.1 cm, being the average of the 3 measurements recorded. The measurements were taken while the subject was standing with feet close together, arms at the side, body weight evenly distributed, and wearing little clothing. In addition, the measurements were taken at the end of a normal expiration and were examined by the same evaluator.

### Statistical analysis

To summarize the main characteristics of the sample, we have used traditional descriptive statistics, and performed the Shapiro-Wilk Normality Test to evaluate the normal distribution of the variables. Student’s T-Test and Mann-Whitney U Test were applied to evaluate differences between groups in parametric and non-parametric data, respectively. A MANCOVA model using Bonferroni’s Multiple Comparison Test was used to verify differences regarding domains of TFEQ and EDE-Q and measures of hunger, appetite and satiety between FM and healthy controls. The potential confounding variables included in the models were leptin levels and BDI score. Pearson’s correlation was used to explore the association of serum biomarkers (BDNF and S100B) as surrogate measures of eating psychopathology in FM. Data were analyzed using SPSS version 21.0 (SPSS, Chicago, IL, USA). For all statistical analysis, significance was set at p < 0.05. The analytic plan was pre-specified, and any data-driven analyses were clearly identified and discussed.

## RESULTS

The demographic and clinical characteristics are shown in Table 1 .

**Table 1:** Demographic and clinical characteristics (n = 39)

		Control	Fibromyalgia	p
		(n = 19)	(n = 20)	
**Demographic**					
Age ° (years)		50 (40-55)	50.5 (46-55)	0.92
Body weight * (Kg)		71.2 (±12.6)	69.8 (±13.1)	0.73
Height * (m)		1.63 (±0.6)	1.59 (±0.7)	0.10
BMI ¢ * (kg/m^2^)		26.9 (±4.6)	27.5 (±4.7)	0.67
Waist perimeter ° (cm)		94.5 (85-104)	92.2 (84-105)	0.81
Level of education * (years)		15 (±4.7)	11.3 (±4.9)	0.02
International Physical Activity Questionnaire – IPAQ-L					
	MET € walking ° (min/week)	528 (248-1617)	742.5 (107-1448)	0.75
	MET € moderate ° (min/week)	840 (360-1540)	495 (135-2055)	0.60
**Clinical**					
Beck Depression Inventory – BDI – II °		2 (1-7)	26.5 (19-34)	<0.001
BPCSI £ *		20.0 (±10.1)	57.3 (±13.7)	<0.001
Pittsburgh Sleep Quality Index – PSQI °		3 (2-5)	11.5 (9-14)	<0.001
**Psychotropic medications**					
Antidepressants					
	Selective serotonin reuptake inhibitors	8/11	-	
	Tricyclic antidepressants	8/11	-	
Anticonvulsants		6/13	-	
Opioids		1/18	-	
Antianxiety agents		1/18	-	
**Biochemical**					
Serum BDNF *		23.7 (±6.2)	27.5 (±4.1)	0.029
Serum leptin *		38.9 (±17.0)	32.5 (±15.5)	0.22
Serum S100B *		28.4 (±5.8)	36.0 (±14.6)	0.042
Serum estradiol °		5 (5-36)	5 (5-58)	1.00

¢ Body mass index.

€ Metabolic equivalent of task.

£ Brazilian Portuguese Central Sensitization Inventory.

* Parametric data were assessed by Student’s T test for independent samples; values represented by mean and standard deviation (SD).

° The Mann-Whitney U test was performed in non-parametric data; values represented as median and P25-P75.

### Multivariate Analysis of disordered eating symptomatology and measures of hunger, feeling of hunger, satiety and appetite for specific and unspecific foods

Table 2 shows the multiple dependent variables in the MANCOVA model, including emotional eating, uncontrolled eating, cognitive restraint, restraint, eating concern, weight concern and body shape concern, hunger scores, feeling of hunger scores, appetite for unspecific foods, appetite for sweet foods, appetite for savory foods and satiety scores according to FM and healthy controls, and serum leptin levels and BDI score, as an independent variable. A MANCOVA model using Bonferroni’s Multiple Comparison Test revealed a significant relationship between the FM group and the outcomes related to dysfunctional eating behavior as assessed by emotional eating [46.0 (±29.5) *vs* . 31.9 (±25.6); *p* = 0.015)], eating concern FM [(0.9 (±1.2) *vs* . 0.2 (±0.3); *p* = 0.044)], weight concern [1.9 (±1.8) *vs* . 0.9 (± 0.8); *p* = 0.007)], shape concern [2.8 (±1.7) *vs* . 1.2 (±0.8); *p* = <0.001], feeling of hunger [5.2 (± 2.9) *vs* . 4.8 (± 2.0); *p* = 0.042] and satiety scores [7.0 (± 1.7) *vs* . 8.3 (± 1.0); *p* = 0.038] (Hotelling’s Trace = 1.522, F = 2.391, P < 0.033). This analysis presented a power of 0.866. The variables included in the model explain above 14% of the variance in the outcome variables. The results of this adjusted multivariate model are shown in Table 2 .

**Table 2:** Eating Psychopathology (n = 39)

Dependent Variables	Type III Sum of Squares	df	Mean Square	F	p	Partial Eta Squared
**Three Factor Eating Questionnaire Domains – TFEQ**						
Cognitive Restraint – TFEq	430.556	3	143.519	0.473	0.703	0.039
Uncontrolled Eating – TFEq	2179.184	3	726.395	2.448	0.080	0.173
Emotional Eating – TFEq	7698.439	3	2566.146	3.975	0.015	0.254
**Eating Disorder Examination Domains – EDEq**						
Restraint – EDEq	10.877	3	3.626	2.712	0.060	0.189
Eating Concern – EDEq	7.186	3	2.395	2.988	0.044	0.204
Shape Concern – EDEq	39.291	3	13.097	8.205	0.000	0.413
Weight Concern – EDEq	24.013	3	8.004	4.772	0.007	0.290
**Hunger, Satiety and Appetite rated by a Numerical Rating Scale**						
Feeling of hunger	49.446	3	16.482	3.043	0.042	0.207
Hunger scores	31.772	3	10.591	1.399	0.260	0.107
Satiety Scores	20.008	3	6.669	3.115	0.038	0.211
Appetite to unspecific foods	50.616	3	16.872	1.870	0.153	0.138
Appetite to sweet foods	25.982	3	8.661	0.777	0.515	0.062
Appetite to savory foods	28.073	3	9.358	1.751	0.175	0.131

Model adjusted to BDI scores and serum leptin levels.

The adjusted determination coefficient of this model (R2) was 0.19, 0.14, 0.36, 0.23, 0.14 and 0.14 for emotional eating, eating concern, shape concern, weight concern, and feeling of hunger and satiety scores, respectively.

### Serum Biomarkers: leptin, BDNF and S100B

We also investigated differences in serum levels of BDNF and S100B between FM and the control group, through Student’s T – Test. FM patients compared to healthy controls presented higher levels of serum BDNF and S100B ( Table 1 ).

### Exploratory analysis to assess the association of eating behavior, catastrophizing behavior and biomarkers of brain plasticity (BDNF and S100B)

In the FM group, serum BDNF was negatively associated with hunger (r = - 0.52; *p* = 0.02), while S100B was positively associated with hunger scores (r = 0.463; *p* = 0.004).

**Figure 2:**
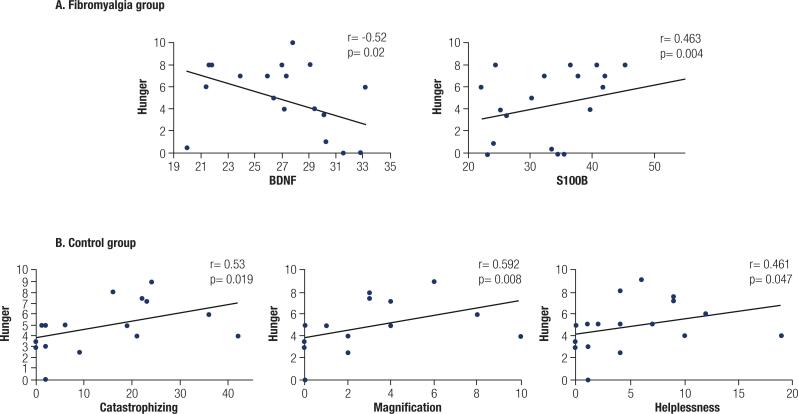
Correlations of eating behavior, catastrophizing behavior and biomarkers. Scatter plots of eating behavior, catastrophizing behavior and biomarkers (n = 39). Abbreviations: brain-derived neurotrophic factor (BDNF) and S100 calcium-binding protein B (S100B).

In order to verify the changes in measures related to eating behavior found in FM group, we also tested the association of these variables with pain catastrophizing and its domains (rumination, magnification and helplessness). Interestingly, we only found an association in the group of healthy controls. Hunger was directly associated with pain catastrophizing (r = 0.53; *p* = 0.019), magnification (r = 0.592; *p* = 0.008), and helplessness (r = 0.461; *p* = 0.047) ( Figure 2 ).

The time of the day in which hunger and appetite for sweet foods were less marked differed significantly between FM and healthy controls, respectively: [16:00 (08:00-14:00) *vs* . 09:30 (8:00-14:15); *p* = 0.039] and [16:00 (10:15-19:15) vs. 10:00 (9:00-14:15); *p* = 0.047]. There were no differences between groups regarding the time of the day in which measures of hunger and appetite were higher.

## DISCUSSION

According to our hypothesis, the results suggest that FM patients presented marked symptomatology of disordered eating, evidenced by higher levels of emotional eating, weight and shape concerns compared to controls matched by age and waist perimeter. Furthermore, we evidenced that FM patients presented augmented perception about the feeling of hunger and reduced satiety scores in comparison to controls. In addition, in the FM group, we found an inverse correlation between serum BDNF and hunger, and a positive association between S100B and hunger scores. The present findings support the notion that disordered eating, observed in FM, might be related to hedonistic rather than homeostatic pathways.

A previous study without a control group reported disordered eating in fibromyalgia. The authors identified that two subscales of EDE-q, weight and shape concern, were potent mediators of the effect of obesity on depression in patients with Primary Fibromyalgia. Moreover, they found an association between BMI and depression, even after binge eating disorder (BED) and poor sleep score were controlled ( 28 ). In our study, disordered eating concerns in FM patients were perpetuated despite the control of depressive symptoms and serum leptin in comparison to healthy subjects, matched for age and waist perimeter.

Eating behavior and pain processing pathway share amendments in brain areas involved in the emotional and affective reactions, e.g., in the dopaminergic mesolimbic system (including neurons of the nucleus accumbens, amygdala, and hippocampus) ( 29 , 30 ), and in the opioidergic system ( 31 ). Both systems orchestrate the motivational reward pathways, known as “wanting” and “liking” ( 32 ). In addition to the role of nucleus accumbens (NAc) in the reward system, which is related to the liking process modulated by the opioidergic signaling, this structure is also involved in the regulation of descending pain modulatory pathways, which contributes to, at least partially, directly or indirectly modulating the perception of noxious information ( 33 ). Assuming that the current pathophysiology of FM involves central sensitization and impairment of descending pain modulatory pathway and that NAc participates in both the pain and the reward systems, we could create a link between the recognized mitigated inhibitory pain response and the diminished satiety perception found in these FM patients.

Likewise, there was a decline of cortical D2/D3 receptor binding availability in the anterior cingulate cortex (ACC) (the primary cortical area in the salience network) ( 34 , 35 ), which plays a role in the aberrant response of both pain and emotional/affective components in a similar pattern found in patients with eating disorders ( 36 ). These findings might be related to decreased and increased motivation and impulsivity responses, respectively, which suggests a conceivable interplay between FM and disordered eating, sustained by shared common dysfunctional neurobiological mechanisms.

We found a negative association between the serum BDNF and self-reported measures of hunger, which at some level could rely on hedonic measure of motivation to reward. Indeed, the role of BDNF in the mesolimbic pathway has been investigated in both pain syndrome ( 37 ) and obesity ( 38 ) and structural changes have been associated with specific amendments in reward and stress circuit targets related to motivation control ( 39 , 40 ). Also, it is known that that the loss-of-function BDNF variants such as the BDNFVal66Met polymorphism may alter vulnerability to stress through two main systems, the HPA axis reactivity and the reward-associated system, both involved in FM, mood and eating disorders physiopathology, representing a possible integration between these pathologies ( 41 , 42 ).

Thus, the findings of disordered eating observed in FM patients might be explained by dysfunctional mechanisms regarding the dopaminergic system. Although we have not measured dopaminergic activity in this study, preclinical and clinical findings could support the underlying mechanisms.

In addition, we found a positive association between S100B and hunger scores. Since S100B is associated with inflammation and hunger can be a surrogate measure of the homeostatic component of eating behavior, we can hypothesize that both this protein and inflammation are related to the homeostatic domains of eating behavior.

In this study, it was not observed an association between pain catastrophizing and eating behavior measures (i.e. hunger, appetite, and satiety). However, in healthy controls, we found a positive association between pain catastrophizing and eating measurement scores. Contrary to our findings, a previous work has postulated that catastrophizing and anxiety sensitivity significantly mediate the relationship between persistent pain and emotional eating ( 43 ). Nonetheless, it should be noted that those authors used a different tool than the one used in this research. It must be addressed that we used a comprehensive evaluation instrument that encompasses how catastrophizing impacts on pain experience, in the sense that their domains are designed to capture emotional aspects strictly associated with pain. Therefore, methodological aspects related to the pain catastrophizing experience can explain the disagreement between the results.

This study had some limitations. First, the hedonic components related to eating behavior had been assessed by an indirect manner, by self-reported measures of feeling of hunger, appetite for specific and unspecific food (savory and sweets) and satiety perception, and also by the domains of TFEQ. However, these measures provide a useful tool for clinical assessment of hedonic hunger, previously described ( 44 ) as a strong and persistent motivation to consume palatable food even if there is no food deprivation. Second, the dysfunctional eating behavior observed in the FM group could misinterpreted because of other variables, such as disturbed sleep pattern and mood disorders. In particular, the regular medication targeting central nervous system could have confounded the results for hunger, appetite and satiety self-reported scores. Nevertheless, we found that these variables did not confound the results obtained in our multivariate models. The time of disease might also be a confounding factor, since this directly impacts treatment time and severity of comorbidities, however it is a difficult measure to be estimated due to the diagnostic difficulty. In addition, our sample presented a statistical difference in educational levels, which is expected, since chronic widespread pain, is more prevalent in adults with poorer socioeconomic status (usually measured by level of education) ( 45 ). However, this measure also might have an impact in other psychiatric features, such as depressive symptoms, so it should be taken into account ( 46 ).

Furthermore, it should be noted that we carefully controlled potential confounding variables that could have biased our findings (depressive symptoms and serum leptin levels). Moreover, the results of this study need to be cautiously interpreted in virtue of its exploratory design. It would be worthwhile to design similar studies to investigate other chronic pain syndromes, such as myofascial pain syndrome or tension-type headaches, to indorse the findings reported in this study.

Taken together, these findings suggest that FM share some neurobiological issues that drive these patients to a disordered eating, which may actively contribute to the higher prevalence of overweight and obesity as comorbidity. Therefore, a better elucidation of the shared pathophysiological adaptations between these two pathologies is crucial to plan therapeutic approaches based on a global health view. Our findings support the notion that the association between FM and obesity is mediated somehow by a hedonistic pathway, rather than a homeostatic pathway. Further research is needed to confirm our initial findings.
